# Machine learning approach for hemorrhagic transformation prediction: Capturing predictors' interaction

**DOI:** 10.3389/fneur.2022.951401

**Published:** 2022-11-24

**Authors:** Ahmed F. Elsaid, Rasha M. Fahmi, Nahed Shehta, Bothina M. Ramadan

**Affiliations:** ^1^Department of Public Health and Community Medicine, Zagazig University, Zagazig, Egypt; ^2^Neurology Department, Faculty of Medicine, Zagazig University, Zagazig, Egypt

**Keywords:** ischemic stroke, hemorrhagic transformation, machine learning, cerebral microbleeds, NIHSS, infarction size

## Abstract

**Background and purpose:**

Patients with ischemic stroke frequently develop hemorrhagic transformation (HT), which could potentially worsen the prognosis. The objectives of the current study were to determine the incidence and predictors of HT, to evaluate predictor interaction, and to identify the optimal predicting models.

**Methods:**

A prospective study included 360 patients with ischemic stroke, of whom 354 successfully continued the study. Patients were subjected to thorough general and neurological examination and T2 diffusion-weighted MRI, at admission and 1 week later to determine the incidence of HT. HT predictors were selected by a filter-based minimum redundancy maximum relevance (mRMR) algorithm independent of model performance. Several machine learning algorithms including multivariable logistic regression classifier (LRC), support vector classifier (SVC), random forest classifier (RFC), gradient boosting classifier (GBC), and multilayer perceptron classifier (MLPC) were optimized for HT prediction in a randomly selected half of the sample (training set) and tested in the other half of the sample (testing set). The model predictive performance was evaluated using receiver operator characteristic (ROC) and visualized by observing case distribution relative to the models' predicted three-dimensional (3D) hypothesis spaces within the testing dataset true feature space. The interaction between predictors was investigated using generalized additive modeling (GAM).

**Results:**

The incidence of HT in patients with ischemic stroke was 19.8%. Infarction size, cerebral microbleeds (CMB), and the National Institute of Health stroke scale (NIHSS) were identified as the best HT predictors. RFC (AUC: 0.91, 95% CI: 0.85–0.95) and GBC (AUC: 0.91, 95% CI: 0.86–0.95) demonstrated significantly superior performance compared to LRC (AUC: 0.85, 95% CI: 0.79–0.91) and MLPC (AUC: 0.85, 95% CI: 0.78–0.92). SVC (AUC: 0.90, 95% CI: 0.85–0.94) outperformed LRC and MLPC but did not reach statistical significance. LRC and MLPC did not show significant differences. The best models' 3D hypothesis spaces demonstrated non-linear decision boundaries suggesting an interaction between predictor variables. GAM analysis demonstrated a linear and non-linear significant interaction between NIHSS and CMB and between NIHSS and infarction size, respectively.

**Conclusion:**

Cerebral microbleeds, NIHSS, and infarction size were identified as HT predictors. The best predicting models were RFC and GBC capable of capturing nonlinear interaction between predictors. Predictor interaction suggests a dynamic, rather than, fixed cutoff risk value for any of these predictors.

## Introduction

Patients with ischemic stroke are at risk of developing hemorrhagic transformation (HT), which could be defined as bleeding within the infarcted area. HT could be precipitated spontaneously or secondary to anticoagulant or thrombolytic reperfusion therapy for ischemic stroke. The reported incidence of HT in patients with stroke varied widely from 0.6 to 85% (1). HT could develop asymptomatically, detected only by CT or MRI, or symptomatically as evident by the associated worsening of existent neurological/clinical manifestation (2). Reperfusion injury had been proposed as the major pathophysiologic mechanism underlying the development of HT. The incidence of HT in ischemic stroke poses a significant risk of deterioration because extravasation of blood could exaggerate inflammatory reactions and promote the progression of brain damage (3, 4). Therefore, detecting susceptibility to HT could facilitate designing management plans for patients with high-risk.

The interaction among risk factors, metabolic, and signaling pathways is a determinant factor in the pathogenies of ischemic stroke, HT, and related complications. The effect of variable interaction on the outcome could be defined by the variance of the outcome that cannot be explained by the main effect of independent factors alone (5). The interaction between predictors is expected when the slope of the relationship between one predictor and the outcome is dependent on (or a function of) another predictor (6). Therefore, describing the interaction between predictors is important for the proper prediction of the outcome. Machine learning (ML) algorithms became increasingly utilized in stroke diagnosis and outcome prediction (7). ML could be broadly classified into supervised and unsupervised based on whether the training data have labeled or unlabeled outcomes, respectively. Supervised ML could be utilized for classification, whereas unsupervised ML could be used to identify hidden patterns such as clustering or abnormal anomaly within the data. Among the most commonly used supervised ML classifiers are logistic regression classifier (LRC), support vector classifiers (SVC), random forest classifier (RFC), decision tree-based gradient boosting classifier (GBC), and multilayer perceptron classifier (MLPC). Machine learning algorithms were reported to efficiently analyze complex non-linear interactions between variables and were utilized to develop prediction models in a variety of clinical settings (8–10).

The objective of the current study was to determine the incidence and predictors of HT, to evaluate predictor interaction, and to identify the best predicting models taking advantage of the state-of-the-art ML algorithms.

## Patients and methods

### Problem formulation

Hemorrhagic transformation prediction was modeled as a supervised learning problem with the incidence of HT as a binary label and selected patient characteristics as features. A total of 16 features including patient clinical data, laboratory findings, and magnetic resonance imaging (MRI) markers were studied. Feature data were collected at patient admission, whereas determining labels was performed using T2 diffusion-weighted MRI conducted at admission and 1 week after.

### Patients and study design

The current prospective study originally included a total of 360 patients with ischemic stroke of whom six patients died before taking the second MRI and 354 completed the study. Participants were recruited using systemic random technique (every 3rd admission) from stroke and intensive care units (ICU), Zagazig University Hospitals, Egypt from January 2018 to February 2020. Participants were subjected to two T2 diffusion-weighted MRIs, at admission and after 1 week to diagnose HT.

### Inclusion criteria

Any patients with ischemic stroke aged ≥18 years old.

### Exclusion criteria

Any patients who received rtPA or suffered from subarachnoid hemorrhage, intracerebral bleeding or any head injuries, hematological disorders, serious liver or renal impairment, coexistent brain infection, tumor, or congenital malformations.

### Clinical and laboratory features

Thorough general and neurological examinations, including NIHSS, were performed. Clinical risk factors were defined as follows: hypertension (receiving medications for hypertension or blood pressure >140/90 mmHg on repeated measurements), diabetes mellitus (receiving medications for diabetes mellitus, fasting blood sugar ≥126 mg/dL or HbA1c ≥6.5%, or a casual plasma glucose >200 mg/dL), hypercholesterolemia [receiving cholesterol-reducing agents or an overnight fasting cholesterol level ≥200 mg/dL, triglycerides ≥200 mg/dL, or low-density lipoprotein (LDL) cholesterol ≥160 mg/dL], in addition to a history of previous heart disease. Performed laboratory tests included blood glucose level, complete blood count (CBC), platelet count, partial thromboplastin time, liver function tests, renal function tests, lipid profile, and erythrocyte sedimentation rate (ESR).

### MRI variables/markers and imaging protocol

Conventional MR, diffusion-weighted image, and GRET2WI (T2^*^WI) were performed using a 1.5 T MR Scanner (Achieva, Philips Medical System). Images were obtained with the patient in the supine position, and a scout sagittal T1-weighted image was used as a localizer; then, multiple pulse sequences were performed to obtain axial, coronal, and sagittal images. The conventional MR sequences included the following: (1) sagittalT1-weighted image as a localizer (TE 8/TR 500 ms), (2) axial T1W-weighted image (TR148-597/TE2-15 ms), (3) axial and sagittal T2-weighted images (TR4400- 4800/TE110 ms), and (4) axial FLAIR (TR6000/TE120-TI2000). Section thickness was set at 5 mm and a gap of 1 mm. We used a field of view equal to 30 mm in coronal images and 240 mm in axial images. T2^*^WI parameters included (TR/TE 641/23 ms) a flip angle of 20, matrix 512 × 512, with a field of view of 250 mm.

For diffusion-weighted imaging, we used a multisection single-shot spin echo planner imaging sequence (TR/TE/NEX: 4.200/140 ms/I) with diffusion sensitivities b-values set at 0 and 1,000 s/mm^2^ and total acquisition time of 80 s. The reconstructed images were transferred to the workstation for the calculation of apparent diffusion coefficient values (11). Analysis was performed by a radiologist ignorant of the study using the software DICOM viewer (v3.0 /v7.2.0.1; Philips Medical Systems Nederland B.V, Best, Netherlands).

Cerebral microbleeds (CMBs) were defined as black (signal loss) or hypointense rounded areas outside the infarcted areas and with <10 mm in diameter on T2^*^Wl (12). The rating of CMBs was performed using the validated Brain Observer Microbleed Scale (13). Superficial siderosis was defined as gyriform hypointensity without corresponding hyperintense signal on T1-weighted sequences or FLAIR (14). Infarction size was determined by the largest diameter of the lesion (15).

The location of ischemic stroke was determined according to Bamford classification as partial anterior circulation (PAC), total anterior circulation (TAC), lacunar, and posterior circulation (POCS) (16). Etiological classification was performed according to the TOAST criteria (Trial of ORG 10172 in Acute Stroke Treatment) into small, large, cardiac, and undermined (17).

### Label

The incidence of HT was defined as any degree of hyperdensity within the area of low attenuation (18). The type of HT was determined according to ECASS II classification (European Cooperative Acute Stroke Study) into hemorrhagic infarction (HI) and parenchymal hematoma (PH) (19). HI was further classified into petechiae affecting the infarction margin (HI1) and confluent petechiae with no mass effect within the infarct area (HI2). PH was subdivided into PH1 affecting <30% of the infarct area and PH2 affecting more than 30% of the infarct area with mass effect (19).

### Ethical conduct and institutional approval

Informed consent was obtained from all participants or their relatives. Ethical approval was obtained from the local ethics committee of our hospital.

### Statistical analysis

#### Descriptive analysis and feature selection

Clinical, neurological, laboratory, and imaging data were introduced at the initiation (admission) and 7 days later. Continuous and categorical variables were presented as mean ± SD and percentages and were compared using *t*-test and chi-square, respectively. The 354 samples were randomly split (the random seed number was set to 64) into two datasets (177 subjects each) to be used as training and testing datasets. Univariate analysis was performed in the training dataset to describe the frequency distribution of variables among HT positive and negative cases. Variable selection is an important step in model building to produce a parsimonious model with reduced data dimensionality, enhanced interpretability, reduced overfitting, and enhanced generalizability (20, 21). We performed variable selection using a filter-based technique, using the minimum redundancy maximum relevance (mRMRe) algorithm, which is provided by the varrank package in R (22, 23).

The mRMRe algorithm selects variables based solely on the characteristics of input data, independent of any model developing algorithm, and thus is robust to overfitting. In contrast to linear correlation, mRMRe algorithms could measure non-linear relationships and remain invariant under variable inevitable transformations (24).

#### Model development, optimization, and comparison

Logistic regression classifier, SVC, RFC, GBC, and MLPC models were developed using the identified predictors, optimized in the training dataset, and used to predict HT incidence in the testing dataset. Grid search was utilized for hyperparameter tuning for GBC, SVC, MLPC, and LRC, whereas automated Bayesian hyperparameter tuning was utilized for RFC. Models were optimized *via* minimizing loss function to reduce misclassification errors in the training dataset (25). SKlearn built-in log-loss function was used for training GBC, LRC, and MLPC, the squared hinge function was used for training SVC, whereas the Gini function was used for RFC. The log-loss function is defined as *L*_log_(*y, p*) = −(*ylog*(*p*) + (1−*y*)log(1−*p*)), where *L*_log_ is the log loss, *y* is the true label, and *p* is the probability estimate that *y* = 1. The log-loss function will be reduced to −log(1−*p*)*if y* = 0, and −log(*p*)*if y* = 1. The squared hinge loss is defined as


$$L\left(\text{y},\;\hat{\text{y}}\right)=\;\sum_{i=0}^{\text{N}}\;(\max{\left(0,\;1-\;{\text{y}}_{\text{i }}\cdot \;\hat{\text{y}}\right)}^{2},$$

where y and $\hat{\text{y}}$ are the true and predicted labels, respectively.

Random forest classifier was optimized using automated Bayesian hyperparameter tuning provided by the open-source hyperopt Python library in the Python environment (26). Hyperopt utilizes a Tree Parzen Estimator (TPE) algorithm, which is Bayesian optimization algorithm that instead of modeling *p*(*y*|*x*) directly, it models *p*(*x*|*y*) and *p*(*y*), such that


$$p\left(x|y\right)=\{\begin{array}{cc} \ell x\;if\; & y<y^{*}\\ \text{ g}x\;if\; & y\geq y^{*}, \end{array}$$

where *l*(*x*) is the density formed by using the observations {*x*^(*i*)^} such that the corresponding loss *f*(*x*^(*i*)^) is less than y^*^ and g(*x*) is the density formed by using the remaining observations (27). Supplementary Table 1 lists the tuned hyperparameters of different models.

The area under the ROC curve (AUC) was used for the evaluation and comparison between models using Delong's non-parametric method (28, 29). AUC is equivalent to the probability of a classifier to correctly discriminating and ranking positive instances higher than negative instances in a randomly chosen sample, which renders it equivalent to the Wilcoxon test of ranks (28). AUC is a prevalence- and threshold-independent metric that was reported to be more robust to data imbalance than accuracy (30). To guard against overfitting, the Youden index identified in the training dataset was used as the classification threshold for ROC analysis in the testing dataset. The model predictive performance metrics were calculated as follows: sensitivity (TP/TP + FN), specificity (TN/TN + FP), predictive positive value (TP/TP + FP), and predictive negative value (TN/TN + FN).

To better understand the relationship between predictor variables and HT incidence, the model 3D hypothesis spaces within the true feature space were plotted. The distribution of HT positive and negative cases relative to the 3D hypothesis space was examined. The interaction of predictors was investigated by fitting a series of the generalized additive model (GAM) with interaction terms, smooth plate regression splines, and tensor product splines using the generalized additive model provided by the mgcv package in the R environment (23, 31). The Python scikit-learn library, version 0.23.2, was utilized to train and test machine learning models using Jupyter Notebook (32, 33). Predicted hypothesis spaces were produced using plotly functions within the Python environment, version 4.10 (34).

#### Sample size

The incidence of HT in patients with ischemic stroke was reported to be around 8.7–12.3% (18, 35). A sample of 126 was estimated to detect the incidence of 9 ± 5% precision with a 95% confidence level and power of 80%. Because we intended to split the sample into training and testing datasets and to compensate for any lost cases, we enrolled 360 participants, of whom 354 continued the study.

## Results

### Patient characteristics, HT incidence, and predictors

Fifty-seven patients were excluded from participation. The main causes were refusal to participate (11), received rtPA (14), head injuries (6), and hematological/liver disorders (26). The clinical and laboratory characteristics of the 354 patients with ischemic stroke who completed the study are presented in Table 1. There was no significant difference between the training and testing datasets except for the NIHSS and PTT. The training dataset demonstrated lower average NIHSS scores and higher average PTT levels compared to the testing dataset. This difference did not influence variable selection because NIHSS, which was lower in the training dataset, was selected and not the higher level PTT. There was no significant difference between the training and testing datasets regarding stroke etiology and location. The percentages of small vessels, large vessels, cardiac, and undermined lesions were 27.1, 33.3, 30.5, and 9.0% in the training dataset and 24.3, 37.3, 28.2, and 10.2% in the testing dataset, respectively. The percentages of lesion PAC, TAC, lacunar, and POCS were 36.7, 18.1, 25.4, and 19.8% in the training dataset and 41.8, 16.9, 24.9, and 16.4% in the testing dataset, respectively. The average duration between stroke onset and ICU presentation was 7.8 (±2.11) in the training dataset, which was not significantly different from that in the testing dataset 7.5 (±2.05).

**Table 1:** Baseline characteristics of patients with ischemic stroke in the total sample and the randomly divided subgroups, the training and testing datasets.

**Characteristic**		**Total sample** **(*N* = 354)**	**Training dataset** **(*N* = 177)**	**Testing dataset** **(*N* = 177)**
Age		62.8 (±10.5)	62 (±11)	63 (±10)
Gender (male)		199 (56.2%)	98 (55.4%)	101 (57.1%)
Hypertension		201 (56.8%)	102(57.6%)	99 (55.9%)
Diabetes mellitus		134 (37.9%)	72 (40.7%)	61 (34.5%)
Smoking		70 (19.8%)	33 (18.6%)	37 (20.9%)
Dyslipidemia		135 (38.1%)	70 (39.5%)	65 (36.7%)
IHD		113 (31.9%)	62 (35.0%)	51 (28.8%)
Platelets		242.89 (±68.9)	241.8 (±71.2)	243.9 (±66.6)
PTT		30.6(±5.5)	31.3 (±6.1)^*^	29.9 (±4.6)
Creatinine		0.73 (±0.2)	0.75(±0.2)	0.72 (±0.2)
Previous stroke/TIA		49 (13.8%)	23 (13.0%)	26 (14.7%)
Antiplatelet		97 (27.4%)	55 (31.1%)	38 (21.5%)
Infarction size		2.47 (±1.8)	2.43(±1.8)	2.5 (1.9)
NIHSS		13.16 (±6.1)	11.7 (±5.4)	13.6 (±6.2)^±^
CMB		2.97 (±4.7)	3.2(±4.9)	2.8 (±4.5)
Superficial siderosis		54 (15.3%)	33 (18.6%)	21 (11.9%)
Duration form symptoms to presentation (hours)		7.7 (2.08)	7.8 (2.11)	7.5 (2.05)
TOAST	Small	91 (25.7%)	48 (27.1%)	43 (24.3%)
	Large	125 (35.3%)	59 (33.3%)	66(37.3%)
	Cardiac	104 (29.4%)	54 (30.5%)	50 (28.2%)
	Undetermined	34 (9.6%)	16 (9.0%)	18 (10.2%)
Location	PACS	139 (39.3%)	65 (36.7%)	74 (41.8%)
	TACS	62 (17.5%)	32 (18.1%)	30 (16.9%)
	Lacunar	89 (25.1%)	45 (25.4%)	44 (24.9%)
	POCS	64 (18.1%)	35 (19.8%)	29 (16.4%)
HT incidence		70 (19.8%)	40 (22.6%)	30 (16.9%)
ECASS II	HI1	20 (5.6%)	13 (7.3%)	7 (4.0%)
	HI2	27 (7.6%)	15 (8.5%)	12 (6.8%)
	PH1	15 (4.2%)	8 (4.5%)	7 (4.0%)
	PH2	8 (2.3%)	4 (2.3%)	4 (2.3%)

* Significantly larger than that of testing dataset.

± Significantly larger than that of training dataset.

Table numbers represent frequencies (%) or means (± SD), which were compared using chi-square and two-sided *t*-test, respectively. The training and testing datasets were not significantly different except for PTT and NIHSS. This difference did not influence variable selection from the training dataset as evidenced by mRMR selection of the lower NIHSS and not the higher level PTT as the informative predictor of HT incidence.

IHD, ischemic heart diseases; PTT, partial thromboplastin time; NIHSS, National Institutes of Health Stroke Scale; CMB, cerebral microbleeds; TOAST, Trial of ORG 10172 in Acute Stroke Treatment; PACA, partial anterior circulation syndrome; TACS, total anterior circulation syndrome; POCS, posterior circulation syndrome; ECASS, European Cooperative Acute Stroke Study for hemorrhagic transformation classification; HI1, hemorrhagic infarction 1; HI2, hemorrhagic infarction 2; PH1, parenchymal hematoma 1; PH2, parenchymal hematoma 2.

The incidence of HT in our ischemic stroke cohort was 19.8% (70 out of 354 patients). No significant difference between the training and testing datasets was observed regarding HT type. The percentages of HI1, HI2, PH1, and PH2 were 7.3, 8.5, 4.5, and 2.3% in the training dataset and 4.0, 6.8, 4.0, and 2.3% in the testing dataset, respectively.

**Figure 1:**
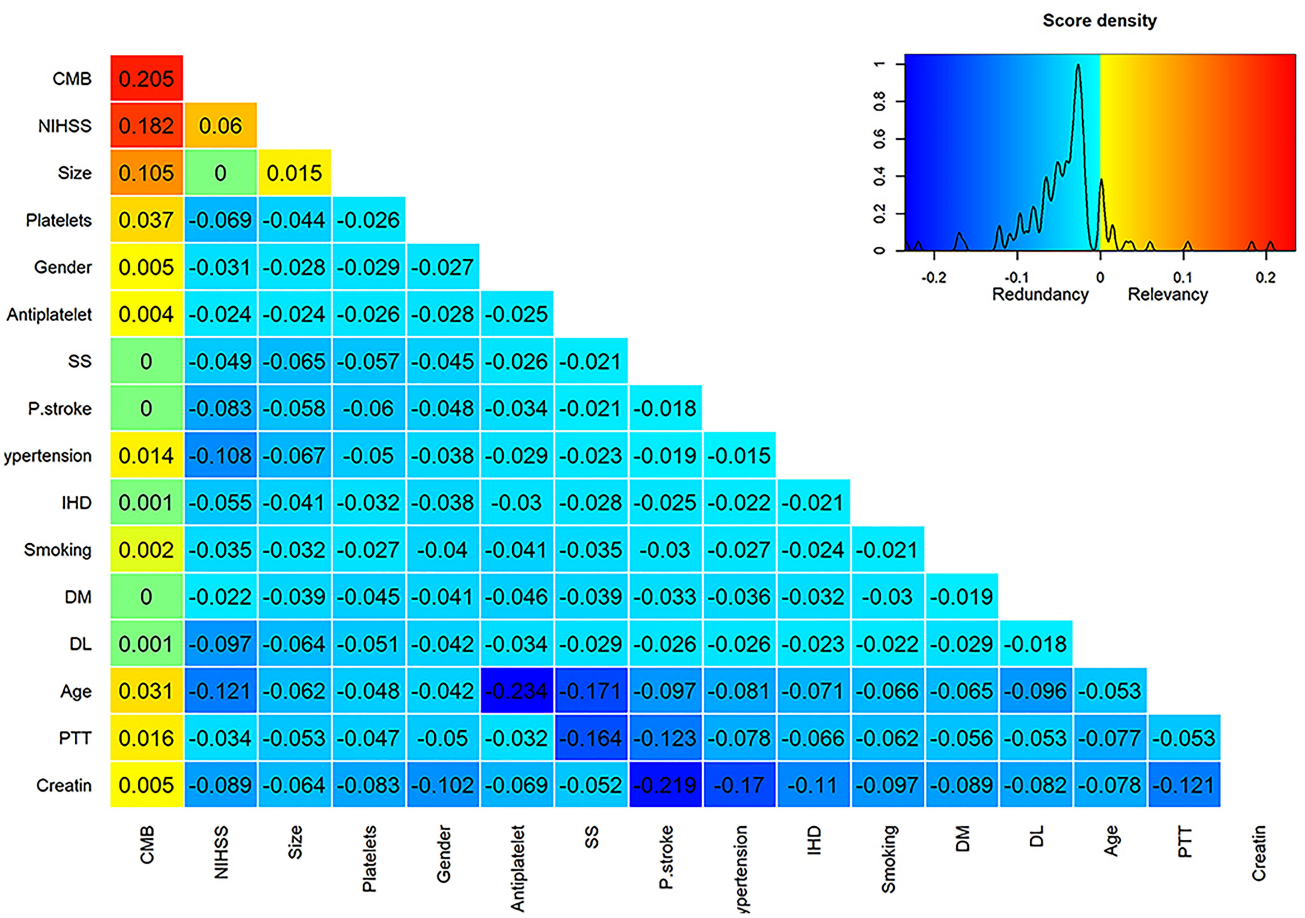
Score matrix of all studied variables assessed by the mRMRe (minimum redundancy maximum relevance) algorithm provided by the varrank R package. The first column represents the relevance scores of different variables assessed by the mutual information algorithm relative to the HT incidence and ranked in a descending manner. Subsequent columns represent the difference between the relevance and redundancy scores of each variable after adding it to the previously selected variable. Positive scores indicate higher relevance than redundancy scores and were color-coded by a scale from yellow to red, whereas negative scores indicate higher redundancy than relevance scores and were color-coded by a scale from aqua to deep blue. Zero scores were colored green.

Using the mRMR variable selection algorithm in the training dataset, it was possible to identify CMB, NIHSS, and infarction size as the most informative variables (Figure 1), and they were used to build HT prediction models. Univariate analysis between HT positive and negative cases in the training dataset confirmed the results obtained by mRMR (Supplementary Table 2). The only significant difference was observed for CMB, NIHSS, and infarction size.

### Comparison of HT predicting models

The learning curves of different models are presented in Supplementary Figure 1. Learning curves present mean accuracy scores (±1 SD) as a function of the sample size used for cross-validation. The learning curve of GBC exhibited an overfitting pattern to the training dataset. However, the performance of GBC in the testing dataset was satisfactory. For RFC and SVC, the learning curves were not significantly lower in the testing dataset compared to the training dataset. For sample sizes >80, RFC and SVC did not exhibit major signs of bias or overfitting as suggested by the difference between the training and testing datasets. The learning curves of LRC and MLPC seem to get closer to accuracies lower than RFC, GBC, and SVC. Also, LRC and MLPC curves exhibited higher variability as suggested by the apparently larger ±SD intervals and crossing of training and testing curves, which may explain their low AUC metrics.

**Figure 2:**
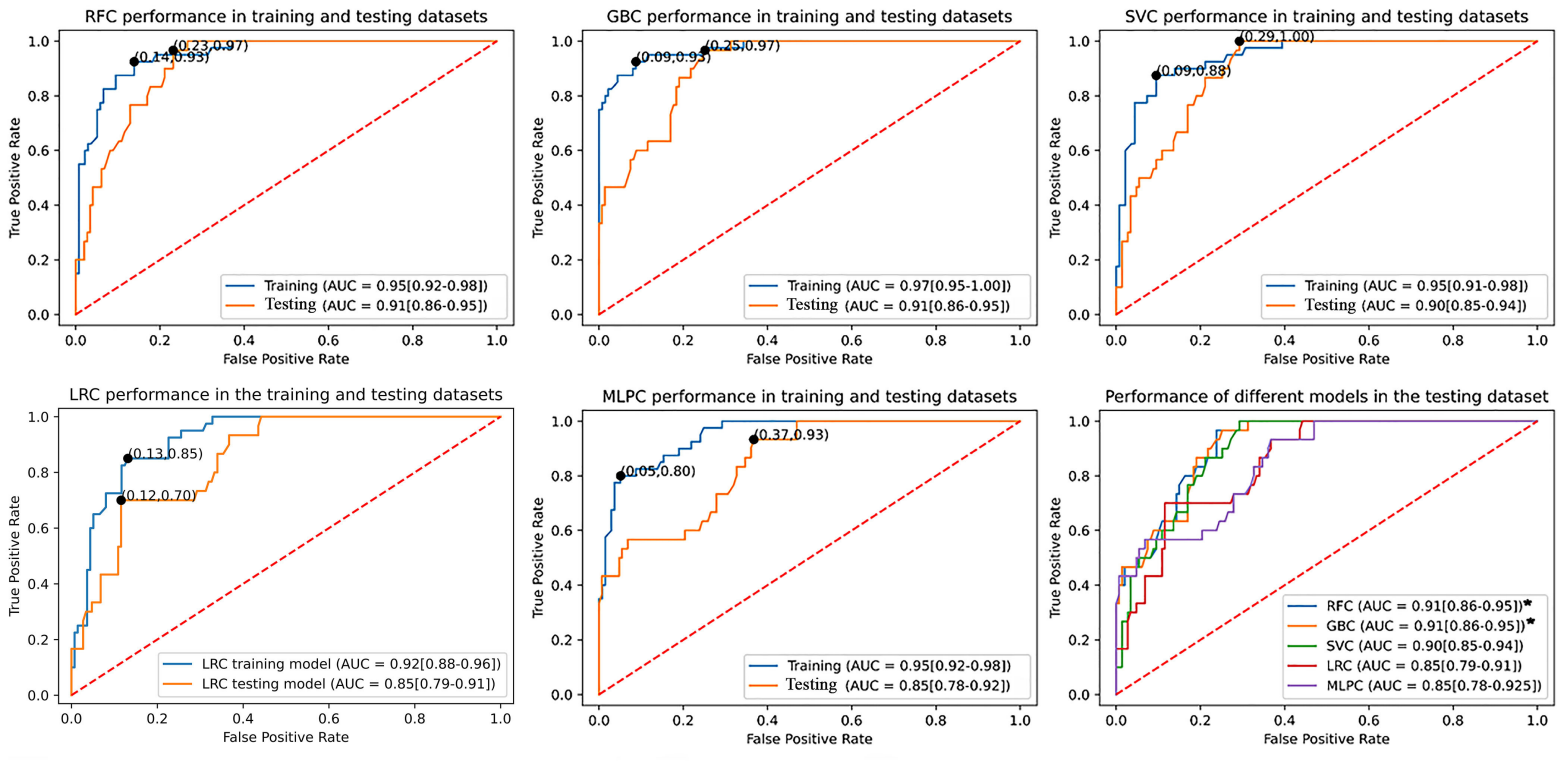
Comparison of the utilized machine learning models' overall performance using the AUC ± 95% CI metric. Youden indices were estimated using the maximum sensitivity plus specificity. The RFC and GBC models demonstrated significantly larger AUC compared to LRC and MLPC but with no statistical difference between each other and SVC. AUC, area under curve; CI, confidence interval; SVC, support vector classifier; GBC, gradient boosting classifier; LRC, logistic regression classifier; RFC, random forest classifier; MLPC, multilayer perceptron classifier.

Table 2 summarizes the performance metrics in the testing dataset. RFC (AUC: 0.91, 95% confidence interval (95% CI): 0.85–0.95) and GBC (AUC: 0.91, 95% CI: 0.86–0.95) demonstrated significantly superior performance compared to LRC (AUC: 0.85, 95% CI: 0.79–0.91) and MLPC (AUC: 0.85, 95% CI: 0.78–0.92). Although SVC (AUC: 0.90, 95% CI: 0.85–0.94) outperformed LRC and MLPC, it did not reach statistical significance. LRC and MLPC did not show significant differences. Figure 2 demonstrates the overall performance of different models in the training and testing datasets as represented by ROC curves. Using classification thresholds determined by the Youden indices identified in the training dataset, the observed classification accuracy in the testing dataset was 0.78 for FRC, 0.76 for GBC, 0.80 for SVC, 0.80 for LRC, and 0.84 for MLPC.

**Table 2:** Predictive performance metrics of different models in the testing dataset.

**Model**	**Sensitivity**	**Specificity**	**PPV**	**NPV**	**AUC (95%CI)^#^**
RFC	0.97	0.74	0.43	0.99	0.91 (0.85–0.95)^*^
GBC	0.97	0.72	0.41	0.99	0.91 (0.86–0.95)^§^
SVC	0.83	0.79	0.45	0.96	0.90 (0.85–0.94)^¥^
LRC	0.70	0.82	0.45	0.93	0.85 (0.79–0.91)^+^
MLPC	0.57	0.89	0.52	0.91	0.85 (0.78–0.92)^+^

# AUC was the metric used for the evaluation and comparison of models performance because it is less sensitive to data imbalance.

* Significantly higher than LRC (*p*-value = 0.021) and MLPC (*p*-value = 0.007) but not GBC (*p*-value = 0.786) and SVC (*p*-value = 0.286).

§ Significantly higher than LRC (*p*-value = 0.021) and MLPC (*p*-value = 0.012) but not SVC (*p*-value = 0.270).

¥ Not significantly higher than LRC (*p*-value = 0.054) and MLPC (*p*-value = 0.056).

+ Was not significantly different from each other (*p*-value = 1).

The MLPC demonstrated very low sensitivity and consequently limited clinical utility and was not considered for 3D model prediction analysis. The classification threshold could be adjusted to modify the misclassification rates (false positive and negative rates) and performance metrics according to the clinical need.

SVC, support vector classifier; GBC, gradient boosting classifier; LRC, logistic regression classifier; RFC, random forest classifier; MLPC, multilayer perceptron classifier; PPV, positive predictive value; NPV, negative predictive value.

To examine whether adding more predictors of HT incidence could improve model performance, we compared the AUC of models developed by all the studied 16 variables to models developed by the mRMR selected three variables. Except for the three-variable SVC model (AUC: 0.90, 95% CI: 0.85–0.94), which significantly surpassed that of the 16-variable model (AUC: 0.82, 95% CI: 0.73–0.90), the AUC of the 16-variable RFC (0.91, 95% CI: 0.87–0.96), GBC (0.91, 95% CI: 0.86–0.95), LRC (0.84, 95% CI: 0.77–0.91), and MLPC (0.85, 95% CI: 0.78–0.92) were not significantly different from those of the three-variable models (Supplementary Figure 2).

### The best performing models 3D predicted spaces suggest the interaction between predictors

**Figure 3:**
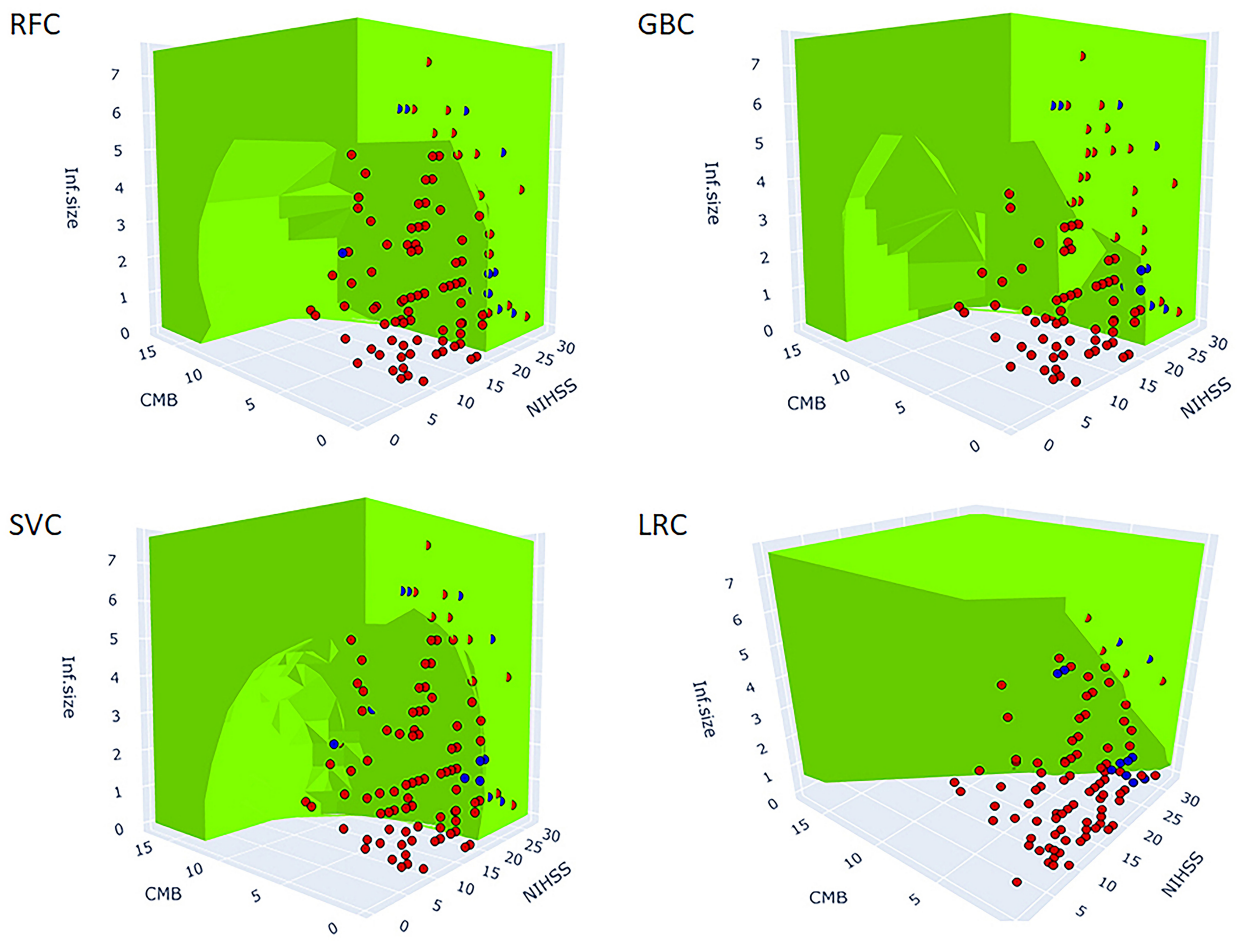
3D figure shows the predicted spaces of each ML model within the true feature space. The green area represents the positive HT prediction, while the non-colored area represents the negative prediction. The blue and red dots represent the observed positive and negative HT cases, respectively (the points inside the green predicted space are not visible). The blue dots within the green and clear areas represent true positive and false negative predictions, respectively. The red dots within the green and clear areas represent false positive and true negative predictions, respectively. The best performing models, RFC, GBC, and SVC reveal non-linear decision boundaries indicative of the interaction between the three predictors. At a particular value of NIHSS, observe the reduction of infarction size needed for HT prediction to be green (positive) as the number of CMB increases. Similarly, at a particular value of CMB, observe the reduction of infarction size needed for HT prediction to be green as the NIHSS score increases. LRC did not capture the non-linear relationships and as such, it failed to model the interaction between predictors. The MLPC was not considered for 3D model presentation because of its very low sensitivity. SVC, support vector classifier; GBC, gradient boosting classifier; LRC, logistic regression classifier; RFC, random forest classifier; MLPC, multilayer perceptron classifier.

Figure 3 shows the 3D predictive space of different models relative to the true feature space. The prediction space of the superior models, RFC, and GBC clearly demonstrated a non-linear relationship and plausible interaction between the predictor variables. In contrast, the lower performance, LRC, and 3D predicted space demonstrated a linear relationship between predictors. This result suggested that failure to detect a non-linear relationship between variables was associated with lower model performance.

### Non-linear interaction of NIHSS with CMB and infarction size

**Figure 4:**
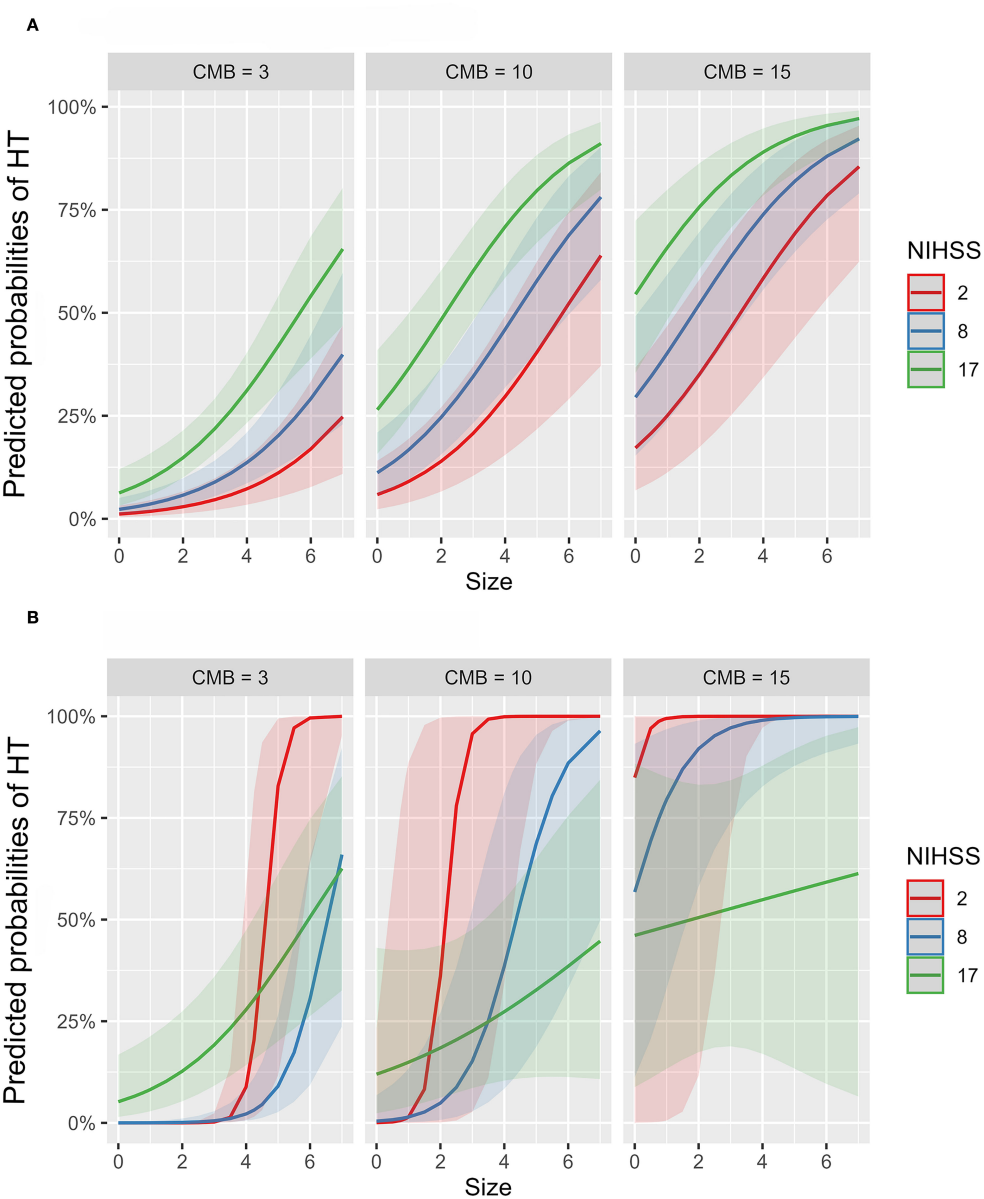
Partial probability of HT incidence as a function of infarction size and conditioned on NIHSS score and CMB count. **(A,B)** show logistic regression fitted with single terms and a generalized additive model fitted with thin plate regression splines with tensor product terms, respectively. Tensor product terms could delineate the interaction component from the main effect. **(A)** shows monotonous NIHSS curves that demonstrate similar parametric functions with infarction size at different CMB levels except for an absolute effect representing different intercepts. This logistic regression pattern suggests a failure to detect predictor interaction and hence predictive power. In contrast, **(B)** demonstrates non-linear HT predicting functions as reflected by NIHSS curves across different levels of CMB and infarction size. For example, at a CMB level of 3, patients with a low NIHSS score of 2 started to respond at an infarction size of around 3.5 and exhibited sharp dependency on infarction size in contrast to patients with an NIHSS of 17, which started to respond at an infarction size of zero but with less dependency on infarction size. At CMB 15, the curve of the NIHSS score of 2 almost reached saturation, whereas the curve of the NIHSS score of 17 almost becomes linear with a low slope demonstrating less dependency on infarction size.

To further investigate the suggested non-linear interaction between HT predictors, we fitted several generalized additive models, all with logit link function, with interaction terms, smooth plate regression splines, and tensor products to the whole dataset. Table 3 demonstrates that adding interaction terms significantly (χ^2^ = < 0.001) improved the performance and resulted in 21 unit reduction of the Akaike information criterion (AIC) score compared to the single-term model. All the coefficients of single and interaction terms were significant. Fitting thin plate regression splines instead of interaction terms has significantly improved model fitting (χ^2^ = < 0.001) and resulted in 16 unit reduction of AIC compared to the interaction term model. Because non-linearity could lead to spurious variable interaction, we fitted models with smoothed predictor and tensor interaction (ti) terms that could delineate the interaction component from the main effect. Fitting product spline significantly improved model performance (χ^2^ = < 0.02) and induced around 3 unit reduction of AIC score compared to models with smoothed splines only. The model revealed the existence of significant non-linear interaction between NIHSS and CMB and between NIHSS and infarction size with effective degrees of freedom (EDF) of 4.6 and 3.1, respectively. Figure 4 shows the partial probability of HT incidence as a function of infarction size and conditioned on NIHSS score (scores of 2, 8, and 17) and CMB (3, 10, and 15). As illustrated in Figure 4A the traditional LR model with single terms produced monotonous NIHSS curves that demonstrate similar parametric functions with infarction size at different CMB levels except for an absolute effect representing different intercepts. This pattern suggests a failure to detect any predictor interaction. In contrast, the model fitted with tensor products, Figure 4B, demonstrated non-linear HT prediction probabilities as reflected by NIHSS curves across different levels of CMB and infarction size. For example, at a CMB level of 3, patients with a low NIHSS score of 2 started to respond at an infarction size of around 3.5 and exhibited sharp dependency on infarction size in contrast to patients with an NIHSS of 17, which started to respond at an infarction size of zero but with less dependency on infarction size. At CMB 15, the curve of the NIHSS score of 2 almost reached saturation, whereas the curve of the NIHSS score of 17 almost becomes linear with a low slope demonstrating less dependency on infarction size. The results presented in Table 3 and Figure 4 lend support to the suggested predictor interaction presented in Figure 3.

**Table 3:** Fitted GAM models to examine predictors linearity and interaction.

**Model**	**Coefficient (SE)**	***p*** **-value**	**EDF**	**Adjusted *R*^2^**	**Deviance**	***p*** **χ2**	**AIC**
**Single term model**							
HT = β_1_CMB + β_2_NIHSS + β_3_ Size	β_1_= 0.24 (0.03)	< 0.001		0.33		Reference	239.4
	β_2_= 0.12 (0.03)	< 0.001					
	β_3_= 0.48 (0.09)	< 0.001					
**Interaction term model**							
HT = CMB + NIHSS + Size + NIHSS*Size + CMB*NIHSS + CMB*Size + CMB*NIHSS*Size	β_1_= 0.98 (0.19)	< 0.001		0.40	29.6^±^	< 0.001^±^	217.9
	β_2_= 0.47 (0.10)	< 0.001					
	β_3_= 1.84 (0.40)	< 0.001					
	β_4_= −0.04 (0.01)	< 0.001					
	β_5_= −0.17 (0.06)	< 0.01					
	β_6_= −0.06 (0.02)	< 0.01					
	β_7_ = 0.007 (0.003)	< 0.05					
**Spline fitted model**							
HT = β_1_CMB + β_2_NIHSS + β_3_Size+	β_1_ = 0.47 (0.2)	< 0.05	1.6	0.50	56.8^±^	< 0.001^±^	201.5
s(CMB) + s(NIHSS) + s(Size)	β_2_ = −0.52 (0.14)	< 0.001	6.8		27.3^§^	< 0.001^§^	
	β_3_ = 1.14 (0.65)	0.08	2.0				
	s(CMB)	< 0.05					
	s(NIHSS)	< 0.001					
	s(Size)	0.09					
**Tensor product model**							
HT= s(CMB)+ s(NIHSS)+ s(Size)+	s(CMB)	< 0.001	2.6	0.52	66.6^±^	< 0.001^±^	197.9
ti(CMB, NIHSS)+ ti(CMB, Size)+	s(NIHSS)	< 0.001	1.0		37.0^§^	< 0.001^§^	
ti(NIHSS, Size)	s(Size)	<0.001	1.0		9.7^¥^	<0.05^¥^	
	ti(CMB,NIHSS)	<0.01	4.6				
	ti(CMB,Size)	0.19	1.0				
	ti(NIHSS,Size)	< 0.001	3.1				

± Compared to single-term model.

§ Compared to interaction term model.

¥ Compared to tensor product model.

Size in the model refers to infarction size. Effective degrees of freedom (EDF) > 1 indicates non-linearity. In the final model, the smooth CMB, the product tensor of CMB and NIHSS, and the product tensor of NIHSS and infarction size were significantly no-nlinear. Intercept was not included in the table.

## Discussion

The objective of the current prospective study was to determine the incidence and predictors of HT, investigate the interaction between predictors, and identify the optimal predicting models. HT incidence in our study was 19.8%, which was in agreement with other studies (35, 36). To produce a parsimonious model with enhanced interpretability, improved accuracy, and enhanced generalizability, we utilized an mRMR algorithm capable of measuring the amount of information between variables (37). It was possible to identify three risk factors namely NIHSS, CMB, and infarction size as potential HT predictors in the training dataset. Detecting the small number of risk factors associated with HT is not unusual. Terruso et al. (38) reported that infarction size was the only significant risk factor associated with HT in their study. Tan et al. (35) found that only infarction size and atrial fibrillation were significantly associated with HT. We could not find sufficient mutual information between HT and age, sex, DM, hypertension, dyslipidemia, platelet count, previous use of antiplatelet, and previous stroke attacks. This finding echoed the results from previous studies (15, 36, 38, 39).

Random forest classifier, GBC, SVC, LRC, and MLPC models were developed using the three identified predictors. The validity of NIHSS, CMB, and infarction size to predict HT incidence was verified by comparison with models developed with all the studied variables. The predictive performance of RFC and GBC models significantly outperformed LRC and MLPC as assessed by the AUC difference. The superior predictive performance of GBC models over classical regression was previously documented in a large study conducted to predict severe complications after acute ischemic stroke (8). In general, the performance metrics of the best ML models in our study were either better or comparable to previous models developed to predict HT incidence in previous studies taking into consideration differences in the utilized HT definition, study design, ethnicity, and sample size of the study group (40, 41).

Infarction size and NIHSS were the most frequently reported risk factors associated with HT either in retrospective or prospective studies (15, 38, 42). Either or both risk factors were commonly included in the previous HT prediction scales in addition to other variables (40–42). CMB was shown by some recent prospective studies to predict the incidence of future hemorrhagic transformation and intracerebral hemorrhage (43, 44). Investigating the optimized model predicted spaces within the true feature/variable space suggested the interaction between predictors as reflected by the non-linear surface as a function of more than one variable. The interaction between predictors was investigated using generalized regression models fitted with smooth splines and tensor products. The results demonstrated the existence of non-linear interaction between predictors and resulted in significant improvement in explaining HT variance compared with models with no interaction terms. This result suggests that there is no fixed cutoff value for any of these predictors after which the risk of hemorrhagic transformation would increase. As shown in Figure 4B, the significance of any of these risk factors to hemorrhagic transformation is changing depending on the values of other risk factors. For example, at a particular value of NIHSS, the value of infarction size needed to increase the risk of HT is reduced if the number of CMB increases. Therefore, the best HT predicting models were those capable of capturing the non-linear relations by algorithms such as RFC and GBC compared to classical LRC.

The interaction implies that NIHSS, CMB, and infarction size could be integrating both similar and distinct upstream pathophysiological mechanisms that collectively could enhance HT incidence. For example, CMB was reported to be significantly associated with advanced age and cerebral small vessel impairment/diseases that occur in hypertension, cerebral amyloid angiopathy, and chronic kidney disease (45–48). The clinical syndromes that have been associated with CMB, such as cognitive impairment, dementia, and recurrent ischemic stroke, further reflect the underlying functional impairment of cerebral small vessels (49). Severe NIHSS score, on the other hand, could integrate risk factors such as old age, female gender, small vessel diseases, and mental and psychological stress of social isolation, and cardiac diseases such as AF, low ejection fraction, heart failure, and cardioembolism. All those risk factors were reported to be significantly associated with NIHSS (48, 50–52).

Several mechanisms could be envisioned to explain the observed interaction between CMB, infarction size, and NIHSS score. One plausible mechanism is *via* cerebral small vessel disease, which could increase both the CMB burden and NIHSS score (48). Cerebral small vessel disease was also reported to be significantly associated with poor collateral recruitment, which could be the underlying mechanism of the observed interaction between CMB, NIHSS, and infarction size. Reduced perfusion from poor collaterals is a determining factor in controlling infarction size, stroke risk, and poor functional outcome (53). Furthermore, cerebral small vessel disease was also reported to be significantly associated with impaired cerebral autoregulation. A growing body of evidence links impaired cerebral autoregulation with the incidence of HT and acute ischemic stroke (54, 55). Genetic susceptibility and APOE ε4 genotype have been reported to be significantly associated with CMB and also with enhanced susceptibility to cerebrovascular insults and, thus, large infarction size (45, 56).

The strength of our study stems from utilizing a prospective study design, using a filter-based, rather than wrapper-based, variable selection technique independent of any model fitting process, and utilizing ML models capable of detecting complex non-linear relationships in a totally naïve testing dataset. In wrapper-based variable selection methods, variables are selected iteratively based on the performance of the fitted model, which could result in models that produce the best performance only with the utilized dataset.

The limitations of our study include the involvement of only one academic hospital and one race. Also, our sample size was relatively small, which could limit the power of our study to detect other risk factors associated with HT incidence. Another source of limitation was the exclusion of patients who received rtPA therapy. Unfortunately, the majority of patients fail to present to our emergency room within the rtPA therapeutic window (57). We thought that the inclusion of those cases could bias results. The death of six subjects before taking the second MRI and hence determination of HT incidence could be a potential source of bias especially if they represented severe cases. In addition, we did not include ASPECTS and collateral scores in our analysis because they were performed for patients on rtPA therapy and not routinely for any patient. The observed moderate PPV suggests the need to include further biological and imaging markers to limit false positive cases. Our results need to be replicated in patients on rtPA therapy to examine the difference in risk profiles and predicting models.

## Conclusion

National Institute of Health stroke scale, infarction size, and CMB were the best HT predictors. The observed interaction between predictors suggests a dynamic, rather than fixed, cutoff risk value for any of these predictors. The best HT predicting models were demonstrated by algorithms capable of capturing non-linear relations such as RFC and GBC compared to the classical LRC. Prediction of HT susceptibility could facilitate designing management plans for high-risk patients.

## Data availability statement

The raw data supporting the conclusions of this article will be made available by the authors, without undue reservation.

## Ethics statement

The studies involving human participants were reviewed and approved by Local Ethics Committee of Neurology Department, Zagazig University Hospitals # 332/2018. The patients/participants or their legal guardians provided their written informed consent to participate in this study.

## Author contributions

AE, RF, NS, and BR: conceptualization and data curation, methodology, and first draft. AE: formal analysis and final version. All authors have read and approved the final manuscript.

## Conflict of interest

The authors declare that the research was conducted in the absence of any commercial or financial relationships that could be construed as a potential conflict of interest.

## Publisher's note

All claims expressed in this article are solely those of the authors and do not necessarily represent those of their affiliated organizations, or those of the publisher, the editors and the reviewers. Any product that may be evaluated in this article, or claim that may be made by its manufacturer, is not guaranteed or endorsed by the publisher.

## Supplementary material

Supplementary Figure 1 Learning curves present mean accuracy scores (±1 SD) as a function of the sample size used for cross-validation. The learning curve of GBC exhibited an overfitting pattern to the training dataset. However, the performance of GBC in the testing dataset was satisfactory. For RFC and SVC, the learning curves were not significantly lower in the testing dataset compared to the training dataset. For sample sizes >80, RFC and SVC did not exhibit major signs of bias or overfitting as suggested by the difference between the training and testing datasets. The learning curves of LRC and MLPC seem to get closer to accuracies lower than RFC, GBC, and SVC. Also, LRC and MLPC curves exhibited higher variability as suggested by the apparently larger ± SD intervals and crossing of training and testing curves, which may explain their low AUC metrics.

Supplementary Figure 2 Area under the ROC curve (AUC) comparison of the three-variable and 16-variable models. Except for the three-variable SVC, which significantly surpassed that of the 16-variable model, the AUC of the 16-variable RFC, GBC, LRC, and MLPC was not significantly different from those of the three-variable models.

Supplementary Table 1 Tuned parameters of five models.

Supplementary Table 2 Univariate analysis of risk factors associated with HT incidence in the training datasets.

## References

[1] Lindley RI Wardlaw JM Sandercock PA Rimdusid P Lewis SC Signorini DF. Frequency and risk factors for spontaneous hemorrhagic transformation of cerebral infarction. J Stroke Cerebrovasc Dis. (2004) 13:235–46. 10.1016/j.jstrokecerebrovasdis.2004.03.003 17903981

[2] Jaillard A Cornu C Durieux A Moulin T Boutitie F Lees KR Hommel M. Hemorrhagic transformation in acute ischemic stroke. The MAST-E study. MAST-E Group. Stroke. (1999) 30:1326–32. 10.1161/01.STR.30.7.1326 10390303

[3] Lei C Wu B Liu M Chen Y. Asymptomatic hemorrhagic transformation after acute ischemic stroke: is it clinically innocuous? J Stroke Cerebrovasc Dis. (2014) 23:2767–72. 10.1016/j.jstrokecerebrovasdis.2014.06.024 25314946

[4] Andrade JBC Mohr JP Lima FO de Carvalho JJF Barros LCM Nepomuceno CR Ferrer JVCC Silva GS. The role of hemorrhagic transformation in acute ischemic stroke upon clinical complications and outcomes. J Stroke Cerebrovasc Dis. (2020) 29:104898. 10.1016/j.jstrokecerebrovasdis.2020.104898 32417239

[5] Lengerich B Tan S Chang CH Hooker G Caruana R. Purifying interaction effects with the functional anova: An efficient algorithm for recovering identifiable additive models. In: International Conference on Artificial Intelligence and Statistics. PMLR (2020). p. 2402–12.

[6] Gennings C Carter WH Jr Carchman RA Teuschler LK Simmons JE Carney EW. unifying concept for assessing toxicological interactions: changes in slope. Toxicol Sci. (2005) 88:287–97. 10.1093/toxsci/kfi275 16081521

[7] Mainali S Darsie ME Smetana KS. Machine learning in action: Stroke diagnosis and outcome prediction. Front Neurol. (2021) 12:734345. 10.3389/fneur.2021.734345 34938254 PMC8685212

[8] Bonkhoff AK Rübsamen N Grefkes C Rost NS Berger K Karch A. Development and validation of prediction models for severe complications after acute ischemic stroke: a study based on the stroke Registry of Northwestern Germany. J Am Heart Assoc. (2022) 11:e023175. 10.1161/JAHA.121.023175 35253466 PMC9075320

[9] Qutrio Baloch Z Raza SA Pathak R Marone L Ali A. Machine learning confirms nonlinear relationship between severity of peripheral arterial disease, functional limitation and symptom severity. Diagnostics. (2020) 10:515. 10.3390/diagnostics10080515 32722280 PMC7459735

[10] Singal AG Mukherjee A Elmunzer BJ Higgins PD Lok AS Zhu J. Machine learning algorithms outperform conventional regression models in predicting development of hepatocellular carcinoma. Am J Gastroenterol. (2013) 108:1723. 10.1038/ajg.2013.332 24169273 PMC4610387

[11] Tong DC Adami A Moseley ME Marks MP. Relationship between apparent diffusion coefficient and subsequent hemorrhagic transformation following acute ischemic stroke. Stroke. (2000) 31:2378–84. 10.1161/01.STR.31.10.2378 11022067

[12] Yates PA Villemagne VL Ellis KA Desmond PM Masters CL Rowe CC. Cerebral microbleeds: a review of clinical, genetic, and neuroimaging associations. Front Neurol. (2014) 4:205. 10.3389/fneur.2013.00205 24432010 PMC3881231

[13] Cordonnier C Potter GM Jackson CA Doubal F Keir S Sudlow CLM. Improving interrater agreement about brain microbleeds: development of the Brain Observer MicroBleed Scale (BOMBS). Stroke. (2009) 40:94–9. 10.1161/STROKEAHA.108.526996 19008468

[14] Charidimou A Jäger RH Fox Z Peeters A Vandermeeren Y Laloux P. Prevalence and mechanisms of cortical superficial siderosis in cerebral amyloid angiopathy. Neurology. (2013) 81:626–32. 10.1212/WNL.0b013e3182a08f2c 23864315

[15] Pan SL Wu SC Wu TH Lee TK Chen TH. Location and size of infarct on functional outcome of noncardioembolic ischemic stroke. Disabil Rehabil. (2006) 28:977–83. 10.1080/09638280500404438 16882637

[16] Bamford J Sandercock P Dennis M Warlow C Burn JJ. Classification and natural history of clinically identifiable subtypes of cerebral infarction. Lancet. (1991) 337:1521–6. 10.1016/0140-6736(91)93206-O 1675378

[17] Chung JW Park SH Kim N Kim WJ Park JH Ko Y. Trial of ORG 10172 in Acute Stroke Treatment (TOAST) classification and vascular territory of ischemic stroke lesions diagnosed by diffusion-weighted imaging. J Am Heart Assoc. (2014) 3:e001119. 10.1161/JAHA.114.001119 25112556 PMC4310410

[18] Paciaroni M Agnelli G Corea F Ageno W Alberti A Lanari A. Early hemorrhagic transformation of brain infarction: rate, predictive factors, and influence on clinical outcome: results of a prospective multicenter study. Stroke. (2008) 39:2249–56. 10.1161/STROKEAHA.107.510321 18535273

[19] Larrue V von Kummer R Müller A Bluhmki E. Risk factors for severe hemorrhagic transformation in ischemic stroke patients treated with recombinant tissue plasminogen activator: a secondary analysis of the European-Australasian Acute Stroke Study (ECASS II). Stroke. (2001) 32:438–41. 10.1161/01.STR.32.2.438 11157179

[20] Dash M Liu H. Consistency-based search in feature selection. Artif Intell. (2003) 151:155–76. 10.1016/S0004-3702(03)00079-132133955

[21] Kumar G Kumar K. An information theoretic approach for feature selection. Sec Commun Netw. (2012) 5:178–85. 10.1002/sec.30335316469

[22] Kratzer G Furrer R. Varrank: An R package for variable ranking based on mutual information with applications to observed systemic datasets. arXiv preprint arXiv:1804.07134. (2018). 10.48550/arXiv.1804.07134

[23] R Core Team. R: A Language Environment for Statistical Computing. R Foundation for Statistical Computing, Vienna (2021). Available online at: https://www.R-project.org/

[24] Tourassi GD Frederick ED Markey MK Floyd CE Jr. Application of the mutual information criterion for feature selection in computer-aided diagnosis. Med Phys. (2001) 28:2394–402. 10.1118/1.1418724 11797941

[25] Jung A. Machine Learning: The Basics. Singapore: Springer (2022). 10.1007/978-981-16-8193-6

[26] Bergstra J Yamins D Cox D. Making a science of model search: Hyperparameter optimization in hundreds of dimensions for vision architectures. In: International Conference on Machine Learning. PMLR (2013). p. 115–23.

[27] Bergstra J Bardenet R Bengio Y Kégl B. Algorithms for hyper-parameter optimization. In:Shawe-Taylor J Zemel RS Bartlett PL Pereira FCN Weinberger KQ, editors. NIPS. (2011). p. 2546–54.

[28] Bradley AP. The use of the area under the ROC curve in the evaluation of machine learning algorithms. Pattern Recognit. (1997) 30:1145–59. 10.1016/S0031-3203(96)00142-2

[29] DeLong ER DeLong DM Clarke-Pearson DL. Comparing the areas under two or more correlated receiver operating characteristic curves: a nonparametric approach. Biometrics. (1988) 44:837–45. 10.2307/2531595 3203132

[30] Ling CX Huang J Zhang H. AUC: a better measure than accuracy in comparing learning algorithms. In: Conference of the Canadian Society for Computational Studies of Intelligence. Berlin, Heidelberg: Springer\ (2003). p. 329–41. Available online at: https://cran.r-project.org/web/packages/mgcv/mgcv.pdf

[31] Wood S Wood MS. Package ‘mgcv'. R package version. (2015) 1:729.

[32] Pedregosa F Varoquaux G Gramfort A Michel V Thirion B Grisel O. Scikit-learn: machine learning in Python. J Mach Learn Technol. (2011) 12:2825–30.

[33] Kluyver T Ragan-Kelley B Pérez F Granger B Bussonnier M Frederic J. Jupyter Notebooks – a publishing format for reproducible computational workflows. In: Positioning and Power in Academic Publishing: Players, Agents and Agendas. Amsterdam: IOS Press (2016). p. 87–90.

[34] Plotly Technologies Inc. Plotly Visualization Library. (2015). Available online at: https://plot.ly

[35] Tan S Wang D Liu M Zhang S Wu B Liu B. Frequency and predictors of spontaneous hemorrhagic transformation in ischemic stroke and its association with prognosis. J Neurol. (2014) 261:905–12. 10.1007/s00415-014-7297-8 24590407

[36] Pande SD Win MM Khine AA Zaw EM Manoharraj N Lolong L. Haemorrhagic transformation following ischaemic stroke: a retrospective study. Sci Rep. (2020) 10:1–9. 10.1038/s41598-020-62230-5 32210323 PMC7093519

[37] Peng H Long F Ding C. Feature selection based on mutual information criteria of max-dependency, max-relevance, and min-redundancy. IEEE Trans Pattern Anal Mach Intell. (2005) 27:1226–38. 10.1109/TPAMI.2005.159 16119262

[38] Terruso V D'Amelio M Di Benedetto N Lupo I Saia V Famoso G. Frequency and determinants for hemorrhagic transformation of cerebral infarction. Neuroepidemiology. (2009) 33:261–5. 10.1159/000229781 19641332

[39] Pundik S McWilliams-Dunnigan L Blackham KL Kirchner HL Sundararajan S Sunshine JL. Older age does not increase risk of hemorrhagic complications after intravenous and/or intra-arterial thrombolysis for acute stroke. J Stroke Cerebrovasc Dis. (2008) 17:266–72. 10.1016/j.jstrokecerebrovasdis.2008.03.003 18755405

[40] Saposnik G Guzik AK Reeves M Ovbiagele B Johnston SC. Stroke prognostication using age and NIH Stroke Scale: SPAN-100. Neurology. (2013) 80:21–8. 10.1212/WNL.0b013e31827b1ace 23175723 PMC3589202

[41] Kalinin MN Khasanova DR Ibatullin MM. The hemorrhagic transformation index score: a prediction tool in middle cerebral artery ischemic stroke. BMC Neurol. (2017) 17:177. 10.1186/s12883-017-0958-3 28882130 PMC5590157

[42] Stone JA Willey JZ Keyrouz S Butera J McTaggart RA Cutting S. Therapies for hemorrhagic transformation in acute ischemic stroke. Curr Treat Options Neurol. (2017) 19:1. 10.1007/s11940-017-0438-5 28130682

[43] Charidimou A Tur G Oppenheim C Yan S Scheitz JF Erdur H. Microbleeds, cerebral hemorrhage, and functional outcome after stroke thrombolysis individual patient data meta-analysis. Stroke. (2017) 48:2084–90. 10.1161/STROKEAHA.116.012992 28720659

[44] Dar NZ Ain QU Nazir R Ahmad A. Cerebral microbleeds in an acute ischemic stroke as a predictor of hemorrhagic transformation. Cureus. (2018) 10:e3308. 10.7759/cureus.3308 32175198 PMC7053796

[45] Poels MM Vernooij MW Ikram MA Hofman A Krestin GP van der Lugt A. Prevalence and risk factors of cerebral microbleeds: an update of the Rotterdam scan study. Stroke. (2010) 41:S103–6. 10.1161/STROKEAHA.110.595181 20876479

[46] Wardlaw JM Smith EE Biessels GJ Cordonnier C Fazekas F Frayne R. Neuroimaging standards for research into small vessel disease and its contribution to ageing and neurodegeneration. Lancet Neurol. (2013) 12:822–38. 10.1016/S1474-4422(13)70124-8 23867200 PMC3714437

[47] Lee J Sohn EH Oh E Lee AY. Characteristics of cerebral microbleeds. Dement Neurocogn Disord. (2018) 17:73–82. 10.12779/dnd.2018.17.3.73 30906396 PMC6428007

[48] Ryu WS Jeong SW Kim DE. Total small vessel disease burden and functional outcome in patients with ischemic stroke. PLoS ONE. (2020) 15:e0242319. 10.1371/journal.pone.0242319 33180837 PMC7660472

[49] Charidimou A Werring DJ. Cerebral microbleeds: detection, mechanisms and clinical challenges. Fut Neurol. (2011) 6:587–611. 10.2217/fnl.11.42

[50] Appelros P Nydevik I Seiger A Terént A. Predictors of severe stroke influence of preexisting dementia and cardiac disorders. Stroke. (2002) 33:2357–62. 10.1161/01.STR.0000030318.99727.FA 12364721

[51] Corso G Bottacchi E Tosi P Caligiana L Lia C Veronese Morosini M. Outcome predictors in first-ever ischemic stroke patients: a population-based study. Int Sch Res Notices. (2014) 2014:904647. 10.1155/2014/904647 27437502 PMC4897223

[52] Lee JY Sunwoo JS Kwon KY Roh H Ahn MY Lee MH. Left ventricular ejection fraction predicts post stroke cardiovascular events and mortality in patients without atrial fibrillation and coronary heart disease. Korean Circ J. (2018) 48:1148–56. 10.4070/kcj.2018.0115 30403019 PMC6221865

[53] Lin L Chen C Tian H Bivard A Spratt N Levi CR. Perfusion computed tomography accurately quantifies collateral flow after acute ischemic stroke. Stroke. (2020) 51:1006–9. 10.1161/STROKEAHA.119.028284 31948385

[54] Castro P Serrador JM Rocha I Sorond F Azevedo E. Efficacy of cerebral autoregulation in early ischemic stroke predicts smaller infarcts and better outcome. Front Neurol. (2017) 8:113. 10.3389/fneur.2017.00113 28392777 PMC5364144

[55] Silverman A Kodali S Sheth KN Petersen NH. Hemodynamics and hemorrhagic transformation after endovascular therapy for ischemic stroke. Front Neurol. (2020) 11:728. 10.3389/fneur.2020.00728 32765416 PMC7379334

[56] Ingala S Mazzai L Sudre CH Salvadó G Brugulat-Serrat A Wottschel V. The relation between APOE genotype and cerebral microbleeds in cognitively unimpaired middle- and old-aged individuals. Neurobiol Aging. (2020) 95:104–14. 10.1016/j.neurobiolaging.2020.06.015 32791423

[57] Bahnasy WS Ragab OA Elhassanien ME. Stroke onset to needle delay: Where these golden hours are lost? An Egyptian center experience. Eneurologicalsci. (2019) 14:68–71. 10.1016/j.ensci.2019.01.003 30671551 PMC6330381

