# Supplementary figures and images for: Machine learning approach for hemorrhagic transformation prediction: Capturing predictors' interaction
Source: Front Neurol. 2022 Nov 24;13:951401. doi: 10.3389/fneur.2022.951401 (PMC9731336; doi:10.3389/fneur.2022.951401)

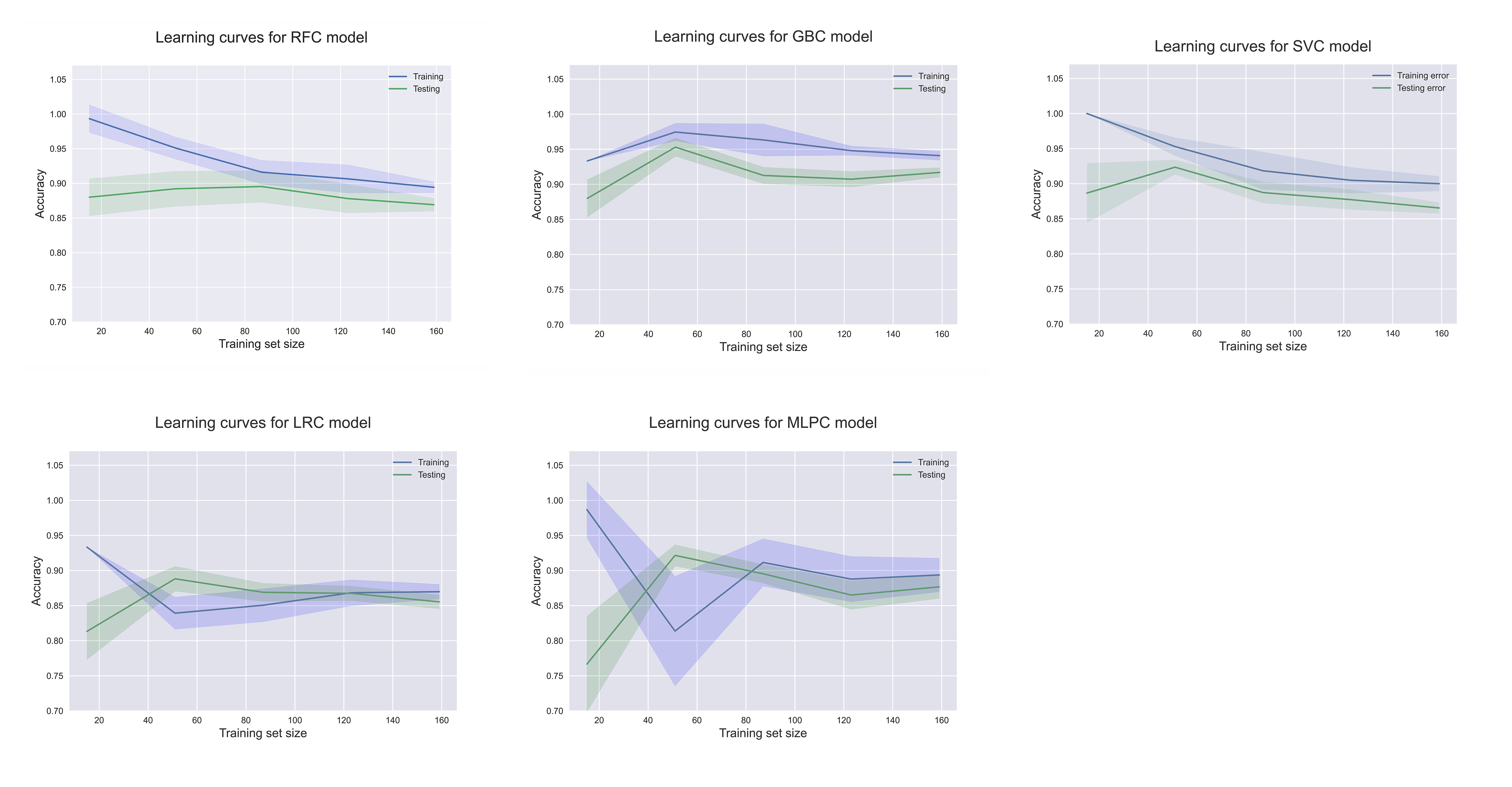

Supplement: Supplementary Figure 1 — Learning curves present mean accuracy scores (±1 SD) as a function of the sample size used for cross-validation. The learning curve of GBC exhibited an overfitting pattern to the training dataset. However, the performance of GBC in the testing dataset was satisfactory. For RFC and SVC, the learning curves were not significantly lower in the testing dataset compared to the training dataset. For sample sizes >80, RFC and SVC did not exhibit major signs of bias or overfitting as suggested by the difference between the training and testing datasets. The learning curves of LRC and MLPC seem to get closer to accuracies lower than RFC, GBC, and SVC. Also, LRC and MLPC curves exhibited higher variability as suggested by the apparently larger ± SD intervals and crossing of training and testing curves, which may explain their low AUC metrics. [file Image_1.tif]

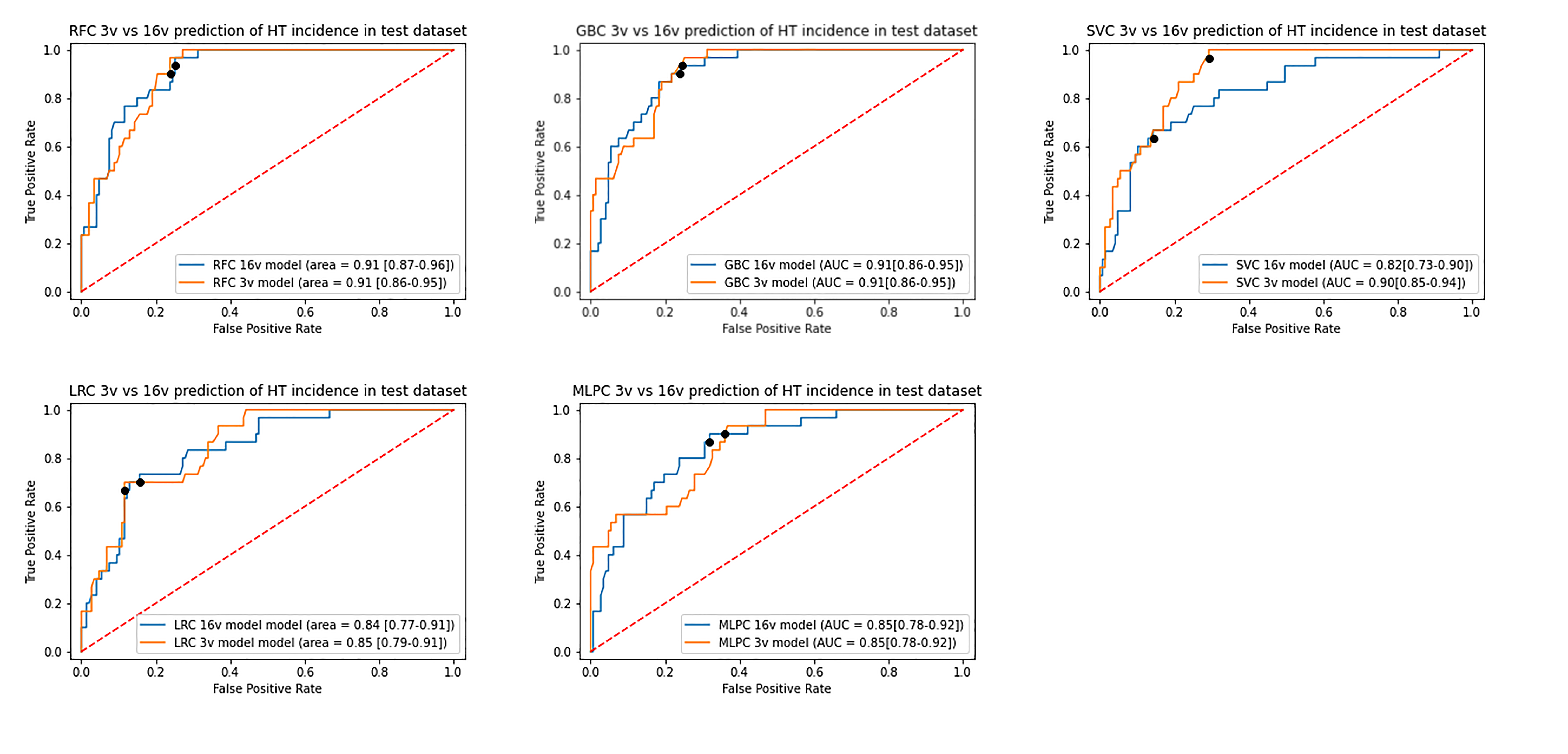

Supplement: Supplementary Figure 2 — Area under the ROC curve (AUC) comparison of the three-variable and 16-variable models. Except for the three-variable SVC, which significantly surpassed that of the 16-variable model, the AUC of the 16-variable RFC, GBC, LRC, and MLPC was not significantly different from those of the three-variable models. [file Image_2.tif]
