# Supplementary material for: Machine learning approach for hemorrhagic transformation prediction: Capturing predictors' interaction
Source: Front Neurol. 2022 Nov 24;13:951401. doi: 10.3389/fneur.2022.951401 (PMC9731336; doi:10.3389/fneur.2022.951401)
Supplement: Supplementary Table 1 — Tuned parameters of five models. [file Table_1.docx]

Tuned parameters of 5 machine learning models

Grid search was utilized for hyperparameter tuning for GBC, SVC, MLPC, and LRC. Automated Bayesian hyperparameter tuning was utilized for RFC.

GBC:

GBC 3v: (learning_rate=0.001, n_estimators=1000,max_depth=4, min_samples_split=6, min_samples_leaf=1,max_features=2 , subsample=0.7)

GBC 16v: (learning_rate=0.1, n_estimators=1000, max_depth=4, min_samples_split=2, min_samples_leaf=1, max_features=4 , subsample=1)

RFC:

RFC3v: {'criterion': 0, 'max_depth': 290.0, 'max_features': 2, 'min_samples_leaf': 0.00096, 'min_samples_split': 0.46293, 'n_estimators': 2}

RFC16v: {'criterion': 1, 'max_depth': 760.0, 'max_features': 2, 'min_samples_leaf': 0.01162, 'min_samples_split': 0.04607, 'n_estimators': 1}

SVC:

SVC3v: parameters = {'kernel':['poly'], 'degree':[2], 'C':[1], 'gamma':['auto'], probability=True }

SVC16v: parameters ={'kernel':['poly'], 'degree':[1], 'C':[100], 'gamma':['auto'], probability=True}

MLPC:

MLPC3v: { solver='adam', activation='relu', alpha=.00001, hidden_layer_sizes=(6, 3, 6), max_iter=1000, verbose= 0, learning_rate_init=0.1}

MLPC16v: {solver='adam', activation='relu', hidden_layer_sizes=(100, ), max_iter=1000, learning_rate_init=0.001}

LRC:

LRC3v: {'C': 0.1, 'max_iter': 100, 'penalty': 'l2', 'solver': 'lbfgs'}

LRC16v: {'C': 0.1, 'max_iter': 100, 'penalty': 'l2', 'solver': 'newton-cg'}
