# Supplementary material for: Machine learning approach for hemorrhagic transformation prediction: Capturing predictors' interaction
Source: Front Neurol. 2022 Nov 24;13:951401. doi: 10.3389/fneur.2022.951401 (PMC9731336; doi:10.3389/fneur.2022.951401)
Supplement: Supplementary Table 2 — Univariate analysis of risk factors associated with HT incidence in the training datasets. [file Table_2.docx]

**Supplementary Table 2.** Univariate analysis of risk factors associated with HT incidence in the training datasets.

| **Characteristic** | | | **HT -Ve**  **(N= 137)** | **HT +Ve**  **(N= 40)** |
| --- | --- | --- | --- | --- |
| Age (years) | | | 62 (±10) | 64 (±12) |
| Gender (Male) | | | 79 (57.7%) | 19 (47.5%) |
| Hypertension | | | 74(54.0%) | 28 (70.0%) |
| Diabetes Mellitus | | | 72 (40.7%) | 61 (34.5%) |
| Smoking | | | 24 (17.5%) | 9 (22.5%) |
| Dyslipidemia | | | 53 (38.7%) | 17 (42.5%) |
| IHD | | | 47 (34.3%) | 15 (37.5%) |
| Platelets | | | 241.8 (±71.2) | 243.9(±66.6) |
| PTT | | | 30.9 (±5.8) | 32.5(±7.2) |
| Creatinine | | | 0.74(±0.2) | 0.75 (±0.3) |
| Previous Stroke/TIA | | | 19 (13.9%) | 4 (10.0%) |
| Antiplatelet | | | 40 (29.2%) | 15 (37.5%) |
| Infarction size | | | 2.1(±1.5) | 3.5 (2.1)* |
| NIHSS | | | 10.5 (±4.4) | 15.8 (±6.6)* |
| CMB | | | 1.8(±3.2) | 7.9 (±6.6)* |
| Superficial siderosis | | | 26 (19.0%) | 7 (17.5%) |
| Stroke onset to presentation (hours) | | | 7.7 (±2.0) | 8.3 (±2.4) |
| TOAST | Small | | 42 (30.7%) | 6 (15.0%) |
|  | Large | | 43 (31.4%) | 16(40.0%) |
|  | Cardiac | | 40 (29.2%) | 14 (35.0%) |
|  | Undetermined | | 12 (8.8%) | 4 (10.0%) |
| Site | PACS | | 53 (38.7%) | 12 (30.0%) |
|  | TACS | | 21 (15.3%) | 11 (27.5%) |
|  | Lacunar | | 38 (27.7%) | 7 (17.5%) |
|  | POCS | | 25 (18.2%) | 10 (25.0%) |
| ECASS II | | HI1 | NA | 13 (32.5%) |
|  |  | HI2 | NA | 15 (37.5%) |
|  |  | PH1 | NA | 8 (20.0%) |
|  |  | PH2 | NA | 4 (10.0%) |

*Significantly larger than that of HT –ve patients.

Table numbers represent frequencies (%) or means (± SD), which were compared using Chi-square and two-sided t-test, respectively. NIHSS, CMB, and infarction size were significantly higher in patients who developed HT compared to negative cases.

IHD; ischemic heart diseases, PTT; partial thromboplastin time, NIHSS; National Institutes of Health Stroke Scale, CMB; cerebral microbleeds, PACA; partial anterior circulation syndrome, TACS; total anterior circulation syndrome, POCS: posterior circulation syndrome, ECASS; European Cooperative Acute Stroke Study for hemorrhagic transformation classification, HI1; hemorrhagic infarction 1, HI2; hemorrhagic infarction 2, PH1; parenchymal hematoma 1, PH2; parenchymal hematoma 2.
